# Safety and Efficacy of Endovascular Therapy for Blood Blister-Like Aneurysms: Willis Covered Stents and Double Stents Assistant Coils—A Single Center Cohort Study

**DOI:** 10.3389/fneur.2021.606219

**Published:** 2021-04-08

**Authors:** Hanxiao Chang, Yuqi Shen, Zheng Li, Chao Lin, Hua Chen, Hua Lu

**Affiliations:** ^1^Department of Neurosurgery, The First Affiliated Hospital of Nanjing Medical University, Nanjing, China; ^2^Department of Neurosurgery, Jiangsu Province Hospital, Nanjing, China

**Keywords:** digital subtraction angiography, embolization procedures, endovascular therapy, blood blister aneurysms, Willis covered stents

## Abstract

**Objective:** To summarize and discuss the application of Willis covered stents (WCSs) and double stent-assisted coils in the treatment of blood blister-like aneurysms (BBAs).

**Materials and Methods:** Thirty-two patients with BBAs treated from January 2015 to October 2020 were included in the study. Among them, 18 were treated using WCSs and 14 using double stents-assisted coils. The indications for treatment, perioperative findings, and postoperative follow-up results were collected and analyzed.

**Results:** All 32 patients had successful stent deployments. Complete aneurysm occlusion was achieved in all 18 patients treated with WCSs immediately. WCS-related adverse events included 2 cases of mild vasospasm and 4 aggressive procedure-related vasospasms during WCS deployment, a case of dissection after WCS deployment, and 1 death due to ipsilateral temporal lobe rebleeding at the sixth day after WCS deployment. In patients treated with double stent-assisted coils, there were 3 cases of neck remnants, 1 acute occlusion of the ipsilateral MCA branch, and 4 mild procedure-related intraoperative vasospasms. The mean follow-up period was 4.2±1.6 months (range 3–6 months). Follow-up imaging data were available for 25 patients (78.1%). In the first postoperative angiographic follow-up, all BBAs were completely occluded. Mild asymptomatic stent stenosis was observed in 3 patients treated with WCSs. Follow-up examination at 6 months after the employment of WCSs showed that the modified Rankin score (mRs) was 0 in 6 patients, 1 in 5 patients, 2 in 3 patients, 3 in 1 patient, 4 in 2 patients, and 6 in 1 patient. After treatment with double stents-assisted coils, the mRs was 0 in 4 patients, 1 in 5 patients, 2 in 3 patients, and 4 in 2 patients.

**Conclusions:** WCSs and double stent-assisted coils for the treatment of BBAs are both safe and efficient. WCSs provide a higher rate of immediate occlusion; however, there was no significant difference in the long term.

## Introduction

Blood blister-like aneurysm (BBA) is a special type of intracranial aneurysm with a relatively low morbidity and high mortality (1–3). Most BBAs are located in the anterior wall or the anterior medial wall of the non-branching sites of the supraclinoid segment of the internal carotid artery (ICA). Their typical clinical manifestation is subarachnoid hemorrhage (SAH). Histologically, BBAs are characterized by the presence of blood clots in the arterial walls, thinned tunica adventitia, absence of collagen in the tunica media, and defective endovascular membranes (4).

These unique morphological and histological features present a challenge to neurosurgeons and neurointerventionalists. The current treatment strategy mainly includes surgical and endovascular methods. The former includes clipping, wrapping, suturing, and isolation coupled with arterial bypass, while the latter includes endovascular ICA ligation, coils, stent-assisted coils, multiple overlapping stents, WCSs, and flow diverters (5).

Although multiple clinical trials have employed various surgical and endovascular techniques, no unified BBA diagnosis and treatment standard has been established due to its complexity. Compared with endovascular methods, microsurgery can provide a higher occlusion rate. However, the disadvantages of microsurgery include the higher rate of complications and neurologic impairments (6–8). Technological improvements in intervention devices have led to improved safety and recovery rates associated with endovascular methods (5). A previous double-center study reported that the rates of complete occlusion and recovery in BBAs when using endovascular treatment were 72.9 and 64%, respectively (3, 9, 10). Owing to the lack of an ideal therapeutic strategy for BBAs, we reported the safety and efficacy of WCSs and double stent-assisted coils in the treatment of BBAs. Herein, we report our strategy and analysis of BBA treatment using endovascular methods.

## Patients

We retrospectively reviewed 32 patients with BBAs treated in ^**^Province Hospital between 2015 and 2020. All patients were treated by WCSs or double stents-assisted coils. The following information on the patients was extracted: patient demographics, aneurysm information (location, size, prior treatment), operation timing, periprocedural complications, and follow-up information. The initial clinical status of the patients was evaluated by the Hunt and Hess grading system and the post operational clinical status of the patients was evaluated by modified Rankin Scale (mRS).

## Diagnosis

The inclusion criteria include (1) aneurysms located at supraclinoid ICA's anterior wall or anterior medial; (2) non-branching sites; and (3) SAH corresponding to the aneurysm. An aneurysm was included as a BBA of the ICA when criteria 1–3 were all matched. Diagnosis results were independently adjudicated by at least two authors.

## Treatment Procedure

All patients had undergone emergency surgeries as early as possible after diagnosis. The duration between operation and the onset of SAH is 2 days in the patients treated by WCSs and 2.2 days in patients treated by double stents assisted coils (*P* = 0.64). Endovascular treatment was performed under general anesthesia using a right femoral approach. After completing cerebral angiography, device type, WCSs or double overlapping stent-assisted coils, was selected. The initial delivery system was a 6F Navien catheter (Ev3) for WCS and a 6F ENVOY catheter (Cordis) for porous stents.

Navien guide catheter was deployed over the neck of the aneurysm to achieve greater support. The stent extended at least 2 mm beyond the neck of the aneurysm on both sides. The diameter of the stent used was ~0.5 mm wider than the diameter of the parent artery. WCS was guided to cross the neck of the aneurysm using a guidewire and deployed with a pressure of 5–6 atmospheres under roadmap guidance after confirming that no branches were influenced repeatedly. If endoleaks were observed, re-expansion of the stent was performed with greater pressure to unfold the stent completely.

ENVOY guide catheter was deployed in the C4 segment of ICA. Stent size was selected according to the largest diameter of the parent artery and the length of the aneurysm. It was ensured that the porous stent extended at least 7 mm beyond the neck of the aneurysm on both sides because at both the ends 2 mm of the stent is unusable. The PLUS (Johnson & Johnson) was guided over the neck for 3 cm by a guidewire that was placed in the middle cerebral artery. The Echelon-10 microcatheter was guided into the aneurysmal sac after condensation molding. Then, half of the first stent was released, and the coil was placed into the aneurysmal sac with the assistance of the stent. After the BBA disappeared, the stent was released completely and the second stent deployed. To enhance the rate of occlusion of the aneurysmal neck, the “lantern technology” was adopted by “pushing and pulling” the LVIS stents.

## Antiplatelet and Anticoagulant Protocols

Once the patient was definitively diagnosed and decided to receive interventional treatment, a dose of 300 mg clopidogrel and 300 mg aspirin was administered through a nasogastric tube before the treatment procedure. During the operation, all patients received intravenous heparin injection to maintain an activated clotting time of 250–300 s. In the case of appearance of fresh stent thrombosis, arterial tirofiban injection was administered through guide catheter as a remedial measure. And intravenous tirofiban injection would last for 8 h. After operation, all patients took dual antiplatelet therapy (clopidogrel 75 mg/day and aspirin 100 mg/day) orally for at least 3 months and single antiplatelet therapy (aspirin 100 mg/day) orally for at least 1 year. On the third day of dual antiplatelet therapy, a thrombelastogram test was performed and suggested the inhibitory rate of AA (arachidonic acid) and ADP (adenosine diphosphate). When the AA inhibitory rate was lower than 50% or the ADP inhibitory rate lower than 30%, adjunctive cilostazol (100 mg, twice a day) was an alternative antiplatelet medicine. For those patients with acute hydrocephalus and altered mental status, external ventricular drainage was immediately performed in the hybrid operating room after neutralizing heparin with protamine.

## Clinical and Angiographic Follow-Up

Results of angiography were categorized using the Raymond Classification as Raymond 1 (complete occlusion: no contrast filling the sack and neck of aneurysm), Raymond 2 (subtotal occlusion: decreased contrast filling the neck of aneurysm), and Raymond 3 (recurrence: contrast filling the sac of aneurysm). On appearance of any uncomfortable symptoms, the patients were made to obtain a brain computerized tomography (CT) or magnetic resonance imaging (MRI) scan. Angiographic and clinical follow-ups were performed for a period of 3–6 months after discharge from the hospital. All patients were assessed using digital subtraction angiography (DSA), and postoperative neurologic disability was evaluated using modified Rankin scale (mRS). Twenty-five patients were evaluated using DSA in our medical center, and their clinical and angiographic data were analyzed. Additionally, MRI once a year would be recommended for those patients with in-stent stenosis.

## Statistical Analysis

Continuous variables are expressed as the mean value ± standard deviation (SD). Categorical variables are presented as the count (percentage). Continuous variables were compared using the unpaired Student's *t*-test. The *p*-values < 0.05 were considered statistically significant.

## Results

This study included 19 men and 13 women, with a mean age of 52.5 years (range, 29–75 years). Among the 32 patients with BBAs, 18 patients were treated using WCSs and others were treated using double overlapping stents-assisted coils. The porous stents including 24 LVISs and 4 EPs (ENTERPRISE) were placed in 14 patients. Demographic information on the patients is provided in Table 1. The preoperative Hunt-Hess grades were I in 1 patient with the deployment of WCSs, II in 7 patients, III in 7 patients, and IV in 3 patients. And the preoperative Hunt-Hess grades were I in 1 patient with the deployment of porous stents, II in 7 patients, III in 5 patients, and IV in 1 patient (*P* = 0.41).

**Table 1:** Endovascular treatment, outcome, and follow-up data for 32 patients with blister-like aneurysms (BBAs).

**Number**	**Aneurysm type**	**diameter/mm**	**Pre- Op H-H**	**Operation Timing (after SAH)**	**Immediate results/** **Raymond**	**Post-op mRS**	**Complications**	**Stents type**
1	L-ICA	4.2*5.2	III	1	1	6	Vasospasm/death	WCS 4.0*10
2	R-ICA	3.7*5.7	IV	3	1	2	/	WCS 4.0*13
3	R-ICA	2.6*3.3	IV	1	1	2	Vasospasm	WCS 4.5*16
4	R-ICA	4.2*5.1	II	2	1	0	Stenosis	WCS 4.5*13
5	L-ICA	3.2*6.6	III	1	1	4	Vasospasm	WCS 3.5*13
6	L-ICA	4.8*5.6	II	1	1	1	Dissection	WCS 4.5*16
7	L-ICA	3.2*3.8	III	3	1	2	/	WCS 4.0*13
8	R-ICA	2.6*3.5	III	6	1	1	Vasospasm	WCS 4.5*16
9	L-ICA	4.5*1.7	II	1	1	0	/	WCS 3.5*13
10	L-ICA	3.1*5.6	III	1	1	1	Stenosis	WCS 4.5*16
11	R-ICA	4.2*5.2	II	2	1	0	/	WCS 4.0*13
12	L-ICA	5.2*5.4	IV	1	1	0	Vasospasm	WCS 4.0*16
13	L-ICA	3.2*2.9	I	2	1	0	/	WCS 4.0*16
14	R-ICA	4.1*3.7	II	3	1	1	/	WCS 4.5*16
15	L-ICA	3.3*4.2	III	2	1	3	/	WCS 4.0*13
16	L-ICA	2.9*3.9	II	1	1	1	Stenosis	WCS 4.0*16
17	R-ICA	1.9*2.7	II	2	1	0	/	WCS 3.5*13
18	L-ICA	2.8*3.5	III	3	1	4	Vasospasm	WCS 4.5*16
19	R-ICA	2.1*4.5	II	2	1	2	/	LVIS 4.5*15
								EP 4.5*27
20	L-ICA	2.7*3.5	III	3	1	1	Vasospasm	LVIS 4.5*20
								LVIS 4.5*20
21	L-ICA	2.4*3.6	III	2	1	1	/	LVIS 4.5*30
								LVIS 4.5*20
22	R-ICA	4.2*6.2	III	1	2	0	/	LVIS 4.5*30
								LVIS 4.5*20
23	R-ICA	4.7*5.2	II	2	1	4	/	LVIS 4.5*30
								LVIS 4.5*30
24	L-ICA	3.4*3.9	II	1	2	1	Vasospasm	LVIS 4.5*30
								EP 4.5*27
25	L-ICA	3.2*3.6	III	1	1	2	MCA thrombogenesis	LVIS 4.5*20
								LVIS 4.5*20
26	L-ICA	3.4*4.6	II	6	1	1	/	LVIS 4.5*20
								EP 4.5*27
27	R-ICA	2.5*3.4	II	3	1	0	/	LVIS 4.5*15
								LVIS 4.5*20
28	R-ICA	2.9*3.8	I	2	1	0	/	LVIS 4.5*15
								LVIS 4.5*20
29	L-ICA	2.6*3.4	II	2	2	1	/	LVIS 4.5*20
								LVIS 4.5*20
30	R-ICA	3.2*3.9	III	3	1	2	Vasospasm	LVIS 4.5*20
								EP 4.5*27
31	L-ICA	3.8*4.1	IV	2	1	3	/	LVIS 4.5*30
								LVIS 4.5*20
32	R-ICA	2.4*3.9	II	1	1	0	Vasospasm	LVIS 4.5*30
								LVIS 4.5*20

In all patients, deployment of the WCSs and porous stents was technically successful. Complete occlusion of the aneurysm was achieved in all 18 patients after the placement of 1 WCS. The procedure of double stents assistant coils resulted in Raymond 1 occlusions in 11 patients (78.6%) and neck remnants (Raymond 2) in 3 patients (21.4%). And 1 acute occlusion of ipsilateral MCA branch and 4 mild vasospasms occurred intraoperationally treated by double stents assistant coils. Six vasospasms happened during the deployment of WCSs and a patient died for ipsilateral temporal lobe rebleeding at 6 days after the deployment of the WCS. A mild dissection was caused by the guiding catheter during the deployment of the WCS. After endovascular therapy, three patients treated by WCS and one patient treated with double stents assistant coils were performed external ventricular drainages immediately due to acute hydrocephalus after neutralizing heparin with protamine in hybrid operation room. And a patient treated by double LVISs assistant coils was performed ventriculoperitoneal shunt after the discontinuation of antiplatelet agents for 3 days in the third month after endovascular therapy due to communicating hydrocephalus. No hemorrhage was detected by CT scans after these invasive operations.

### Follow-Up Results

**Figure 1:**
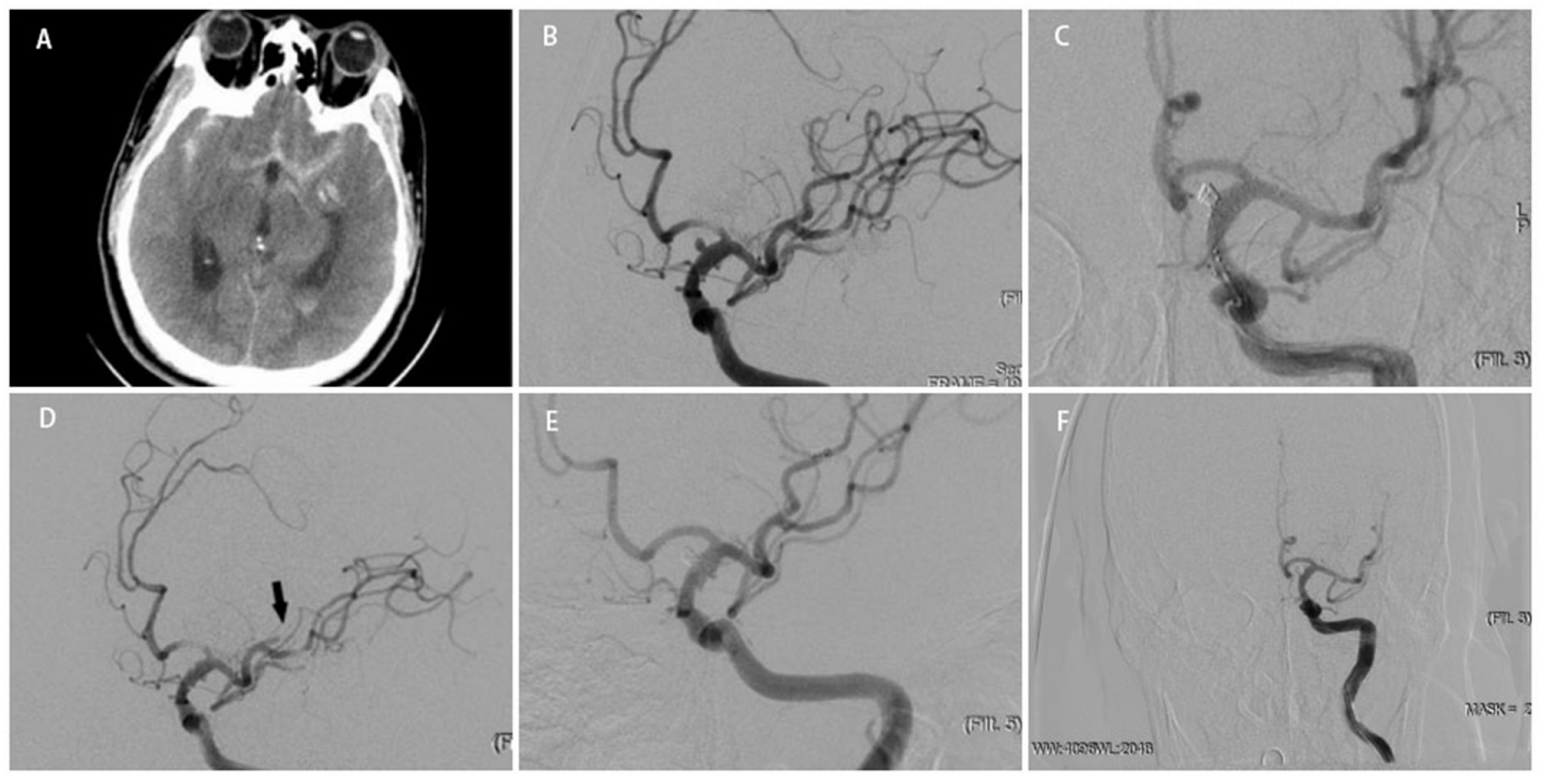
A middle-aged person was admitted to our hospital for sudden headache 10 h earlier (H-H grade 3). An emergency cranial CT revealed diffuse subarachnoid hemorrhage **(A)**. Cerebral angiography at different angles showed a BBA located at the side wall of the C7 segment of right ICA. Multiple stents+coils got the nod to protecting anterior choroidal artery and posterior communicating artery from acute occlusion **(B)**.Two LVIS stents (LVIS 4.5 ^*^ 20 mm) combined with coiling were delivered and deployed successfully, and the instant angiographic result revealed no contrast filling into the aneurysm **(C)**. Intraoperative angiographic revealed that a branch of MCA occluded(black arrow) **(D)**. After tirofiban injection, angiographic revealed the reappearance of MCA branch and total occlusion of the aneurysm **(E)**. Angiographic follow-up at 5 months revealed total occlusion of the aneurysm **(F)**.

**Figure 2:**
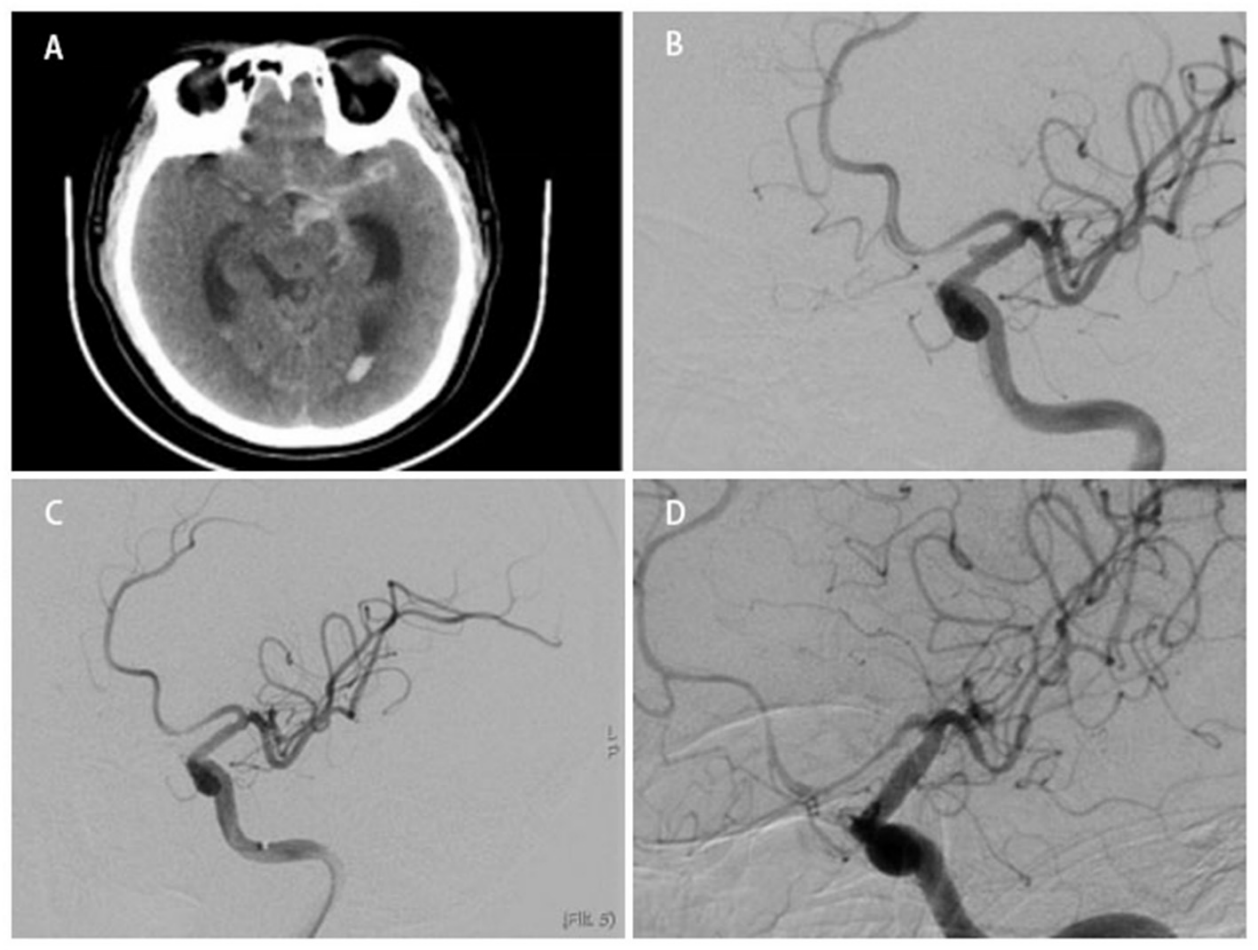
A middle-aged person was admitted to our hospital for sudden headache 8 h earlier (H-H grade 2). An emergency cranial CT revealed diffuse subarachnoid hemorrhage **(A)**. Cerebral angiography showed a BBA located at the side wall of the C7 segment of left ICA **(B)**. After a WCS (3.5 ^*^ 13 mm) deployed successfully, the instant angiographic after once balloon dilation revealed no contrast filling into the aneurysm **(C)**. Angiographic follow-up at 4 months revealed total occlusion of the aneurysm **(D)**.

The mean follow-up period was 4.2 ± 1.6 months (range 3–6 months). Twenty-five patients were available for the imaging follow-ups. All BBAs had been occluded while preserving the patency of the ICA. Additionally, the Raymond 2 occlusions in the 2 patients that had neck remnants improved to Raymond 1 occlusion. And the mild dissection disappeared in the follow-up. However, 3 patients showed in-stent asymptomatic stenosis with the deployment of WCSs and they continued to receive dual antiplatelet therapy orally and were followed up regularly. Follow-up examination after the endovascular treatment showed that good clinical outcome (mRS score of 0–2) were achieved in 14 patients (77.8%) treated by WCSs and 12 patients (85.7%) treated by double stents-assisted coils (*P* = 0.66). Illustractive cases were shown in Figures 1, 2.

## Discussion

With the improvement in endovascular interventional devices, interventional therapy has gradually become mainstream in BBA treatment (5, 11). Despite the lack of a uniform treatment guideline, various interventional treatment strategies have achieved satisfying outcomes, including WCSs, double porous stent-assisted coils, and flow diverters. A former study reported that 76.5% of patients achieved complete aneurysm occlusion and only 9.2% of patients encountered serious complications (12). The use of double porous stent-assisted coils results in higher recurrence and intraoperative rebleeding rates compared with WCSs and flow diverters. However, the use of flow diverters for BBAs is limited in China. Therefore, the use of WCSs and double porose stent-assisted coils are the main treatment strategies in our center. To our knowledge, this is the first study comparing WCSs with double stent-assisted coils in a single clinical setting. In the present study, we reported the comparison of results from the use of WCSs and double porous stent-assisted coils in patients with BBAs.

### Indication

**Figure 3:**
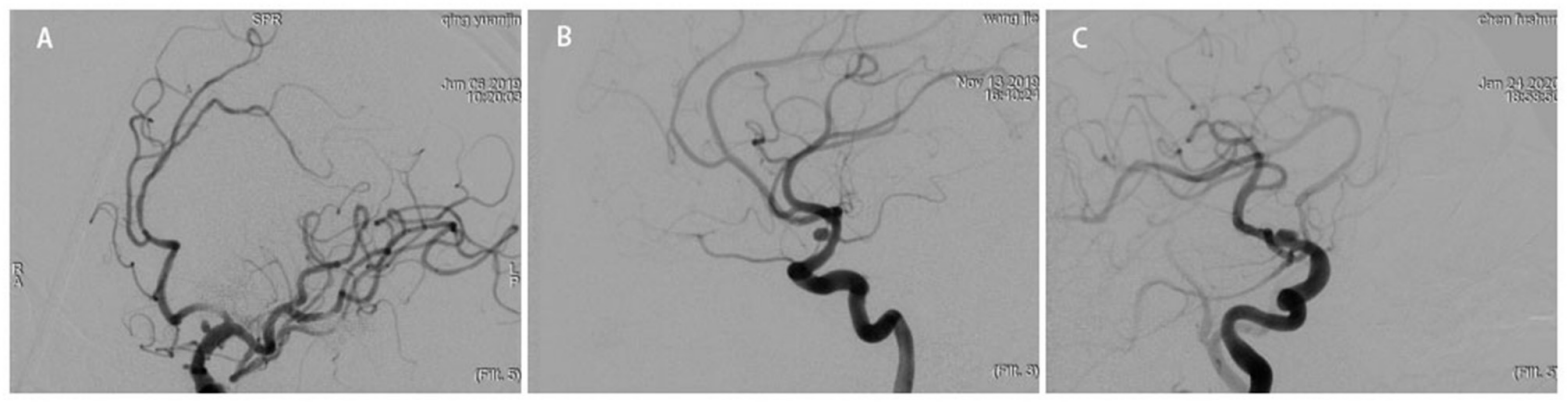
Preoperative digital subtraction angiography (DSA) revealed that the aneurysm was located between the AChA and PCoA **(A)** and in opposite side of the AChA **(B)**. Preoperative DSA revealed that the ICA was too tortuous to use a WCS **(C)**.

Given their unique histological and pathological characteristics, BBAs must be treated as early as possible once definitively diagnosed. Theoretically, covered stents such as WCSs are the first choice due to their ability to block the aneurysmal neck and occlude the aneurysm immediately. In fact, the use of covered stents in clinical practice is strictly restricted due to their design (9). In our medical center, we give priority to the use of covered stents in treatment of aneurysm except in the following three situations: (1) when the clinoid segment of the ipsilateral ICA is too tortuous for WCSs; (2) when the distance between the lesion and the anterior choroidal artery (AChA) is <2 mm; or (3) when the difference between the proximal and distal diameters of the artery is >0.5 mm (Figure 3). Compared with WCSs, porose stents such as LVIS and EP have excellent compliance when dealing with tortuous arteries and are less likely to cause branch occlusion. Despite the poor immediate occlusion rate, the blood flow direction of double overlapping stents guarantees favorable outcomes more often. However, WCSs are restricted to treating aneurysms close to branched vessels. The majority of BBAs are located at the C5–C7 segments of the ICA, where the ophthalmic, anterior choroidal, and posterior communicating artery (PoCA) originate. To avoid infarction, we selected porous stents and coils over WCSs to occlude aneurysms since WCSs work by the plying-up of the polytetrafluoroethylene membrane against the vascular wall. This membrane is easily compromised when deployed inside vessels when the proximal and distal diameters have a difference over 0.5 mm. In this study, we employed ENVOY as a guiding catheter, which is sufficient to support deploying porous stents. For deploying WCSs, we used the Navien catheter. Greater support would accompany with a higher risk of vasospasm and intimal injury.

Despite the above-mentioned limitations, WCSs are still preferred over porous stents for the treatment of BBAs due to the following advantages: (1) haemodynamically, WCSs can rebuild vascular pathways effectively and supply an attachment for the tunica intima; (2) the deployment of WCSs is safe, swift, invasive, and technically simple; (3) WCSs can occlude BBAs immediately, which decreases rebleeding during the perioperative period; and (4) no space-occupying lesions occur after WCSs placement (11, 13, 14). On the contrary, double overlapping porose stents rarely cause side branch acute occlusion and easily pass through tortuous vessels. Surgeons also can achieve a better plying-up by massaging and pushing the porose stents.

### Rebleeding

Owing to the lack of collagen and internal elastic membranes and because of the need to administer antiplatelet and anticoagulant therapy, the perioperative rebleeding rate was higher than in other types of aneurysms, irrespective of the method performed. Even in experienced centers, intraoperative rupture rates in BBAs have been reported to be up to 50% compared with the rate of 7% seen with saccular aneurysms (7). Compared with WCSs, double stent-assisted coils have the possibility of puncturing the aneurysm during the treatment procedure. During the acute phase of treatment, only several patients achieved Raymond 1 occlusion. The remnants of the aneurysmal neck and sac were potential risk factors for rebleeding. Additionally, WCSs can block the blood flow to occlude aneurysms immediately. However, a single patient who had a WCS deployed suffered from cardiac arrest 6 days after surgery and died in the hospital. Immediate computed tomography scans that were performed for this patient showed ipsilateral temporal lobe rebleeding. Upon inspection of the intraoperative images and vital signs, we speculated that an endoleak with relief vasospasm could cause the rebleeding. Regretfully, our conjecture was not proven correct by cerebrovascular imaging.

### Stent Deployment

During our treatment procedures, we transported stents using 6F ENVOY and Navien catheters. The former was positioned into the C4 segment of the ICA and the latter was positioned as close as possible to the lesion. In this study, all 28 porose stents (24 LVISs and 4 EPs) were successfully deployed. There was no arterial dissection and only 4 cases of mild vasospasm occurred, which were alleviated instantly by arterial injection of nimodipine. However, there were 2 cases of mild vasospasm and 4 cases of aggressive vasospasms during the transport of WCSs. The aggressive vasospasms were usually caused by the stimulation caused by WCSs for arterial wall. When aggressive vasospasms occurred, we usually enhanced the support by positioning the Navien catheter as close to the lesion as possible to transport the WCS through the bending blood vessels easily and quickly. After the WCS was in place, nimodipine was injected intravenously until the end of surgery. Two of the cases of aggressive vasospasm were not relieved until the end of the procedure, which may explain the postoperative rebleeding. Liu et al. have reported that about half the patients suffered vasospasm when treating carotid cavernous fistulas with WCSs (15, 16). Except for reversible vasospasm, artery dissections caused by rigid catheters and stents also cannot be ignored, especially when passing through the cavernous sinus segment.

Porose stents proved superior in reducing the infarction rate by completely unfolding in case of tortuous parent arteries. WCS lacked adaptability due to its complex structure, which consists of three parts: a bare stent, an expandable polytetrafluoroethylene membrane, and a balloon catheter. To ensure better wall adherence and reduce endoleaks, shorter stents that expand repeatedly are preferred. However, this may increase the procedural difficulty and the risk of vascular damage. In the present study, 12 aneurysms disappeared after a single balloon dilation and 6 endoleaks occurred. Although the endoleaks were resolved by adjusting and expanding repeatedly, this may cause potential complications, such as intimal injuries and thrombogenesis.

Despite the preoperative administration of antiplatelet and intraoperative anticoagulant therapy, the risk of acute thrombogenesis should still be taken seriously (17). The main risk factors include intimal injury and coagulation reaction from stents and catheters. A former study reported that 12.5% of patients (5 of 40) experienced perioperative thrombosis (18). Incomplete stent apposition should also not be ignored. Repeated and multi-angle angiography can provide immediate evidence of thrombus formation as filling defects or branch occlusions. The possibility of acute thrombus formation increases with longer operative time and poor stent adherence. Therefore, suitable operation planning and stent models are vitally important. In our present study, LVIS stents were used more frequently (24/28) than EPs. The tail of these stents often shows inadequate adherence when placed in a tortuous vessel and a J-shaped microconductance wire can be used to knead the stents repeatedly to unfold completely. Additionally, laser-carving stents such as the EP rarely show incomplete apposition and are a better first choice compared with braided stents (19, 20). The incomplete stent apposition of WCSs can be solved by expanding them repeatedly. A postoperative intravenous tirofiban drip is an appropriate treatment to prevent thrombosis. In the present study, only 1 patient treated by LVIS and coils showed an acute branch occlusion (middle cerebral artery upper branch), which was resolved using tirofiban injected through an arterial catheter. During the postoperative follow-up, 3 cases of asymptomatic stenosis (both stenoses <50%) were detected. However, there were no abnormal detections after thrombelastography testing. The underlying reason for the stenosis could be poor stent adherence and resistance to dual antiplatelet therapy (DAPT) (9, 19). Tan et al. reported that the mean in-stent stenosis rates with WCS deployment at 2 and 6 years were 18.0 and 29.0%, respectively (21). The timing of invasive surgery after antiplatelet therapy remains controversial. Although no hemorrhage was detected in our 4 cases, the risk of procedure-related hemorrhage should be taken seriously. However, Hudson et al. (22) suggested that both antiplatelet-associated hemorrhaging and the timing between external ventricular drain placement and DAPT initiation did not appear to be clinically significant.

### Comparison With Pipeline Embolization Device (PED)

Recently, the application of PEDs in the treatment of intracranial aneurysms has been widely recommended, and BBA embolization by PED is also being attempted. PEDs show good compliance when navigating tortuous arteries, lower risk of branch occlusion, and better complete aneurysm occlusion rate at follow-up evaluations. However, the rebleeding risk in the acute period remains unknown. Mokin et al. (4) noted that although the immediate complete occlusion rate was relatively low, a good clinical outcome was achieved in 68% of patients, complete occlusion was observed in 90% of cases on follow-up angiography, and only 1 case of rebleeding occurred during the perioperative period. Thus, PED is also a safe and effective therapeutic modality for BBAs. Regrettably, the use of PED for BBAs is subject to health insurance policies in our country.

### Study Limitations

This study has several limitations. First, this study did not follow a double-blinded contrast design. The selection of cases was biased because of medical ethics. Second, the number of patients was less and the follow-up period was too short. Third, this study lacks a comparison with flow diversion method because of the restrictions enforced by health care policies.

## Conclusion

Despite achieving 100% immediate occlusion rate, the postoperative mRs scores in patients who were treated with WCSs did not show a clear advantage due to the higher complication rate and increased complication severity.

## Data Availability Statement

The raw data supporting the conclusions of this article will be made available by the authors, without undue reservation.

## Ethics Statement

The studies involving human participants were reviewed and approved by the studies involving human participants were reviewed and approved by Jiangsu Province Hospital. The patients/participants provided their written informed consent to participate in this study. The patients/participants provided their written informed consent to participate in this study.

## Author Contributions

HL and HaC put forward the viewpoint of this article. HaC wrote this report. CL, ZL, and HuC participated in those operation. HL, HaC, and YS collected the data. All authors contributed to the article and approved the submitted version.

## Conflict of Interest

The authors declare that the research was conducted in the absence of any commercial or financial relationships that could be construed as a potential conflict of interest.
